# Dependency of Catalytic Reactivity on the Characteristics of Expanded Graphites as Representatives of Carbonaceous Materials

**DOI:** 10.3390/molecules30112275

**Published:** 2025-05-22

**Authors:** Do Gun Kim, Seong Won Im, Kyung Hwan Ryu, Seoung Ho Jo, Min Gyeong Choe, Seok Oh Ko

**Affiliations:** 1 Department of Environmental Engineering, Sunchon National University, Suncheon 57922, Republic of Korea; swim9828@scnu.ac.kr (S.W.I.); 20184030@s.scnu.ac.kr (S.H.J.); 20204036@s.scnu.ac.kr (M.G.C.); 2 Department of Chemical Engineering, Sunchon National University, Suncheon 57922, Republic of Korea; khryu@scnu.ac.kr; 3 Department of Civil Engineering, Kyung Hee University, Yongin 57922, Republic of Korea

**Keywords:** antibiotics, catalytic degradation, expanded graphite, peroxydisulfate, thermal treatment

## Abstract

Carbonaceous materials (CMs) have gained great attention as heterogeneous catalysts in water treatment because of their high efficiency and potential contribution to achieving carbon neutrality. Expanded graphite (EG) is ideal for studying CMs because the reactivity in CMs largely depends on graphitic structures, and most surface of EG is exposed, minimizing mass transfer resistance. However, EG is poor in adsorption and catalysis. In this study, EG was modified by simple thermal treatment to investigate the effects of characteristics of graphitic structures on reactivity. Tetracycline (TC) removal rate via activating peroxydisulfate (PDS) by the EG treated at 550 °C (EG550) was more than 10 times that of EG. The thermal modification did not significantly increase surfaces but led to increases in damaged, rough surfaces, graphitization degree, C content, defects, and C=O. Radical and non-radical pathways, such as SO_4_^•−^, O_2_^•−^, ^1^O_2_, and electron transfer, were involved in TC removal in EG550+PDS. TC degradation in EG550+PDS was initiated by hydroxylation, followed by demethylation, dehydroxylation, decarbonylation, and ring-opening. The ions ubiquitous in water systems did not significantly affect the performance of EG550+PDS, except for H_2_PO_4_^−^ and HCO_3_^−^, suggesting the high potential of practical applications. This study demonstrated that graphitic structure itself and surface area are not detrimental in the catalytic reactivity of CMs, which is different from previous studies. Rather, the reactivity is governed by the characteristics, i.e., defects and functional groups of the graphitic structure. It is thought that this study provides valuable insights into the development of highly reactive CMs and the catalytic systems using them.

## 1. Introduction

Recently, metal-free CMs have gained great attention as heterogeneous catalysts in the degradation of refractory micropollutants via activating PDS or PMS [1,2]. The CMs include pristine and/or modified CNTs, graphite, gCN, ACs, and biochars, and have gained great attention [1,3,4,5,6]. Importantly, CMs would significantly contribute to carbon neutrality via mitigating greenhouse gas emissions and CO_2_ sequestration [7,8]. CMs are characterized to have excellent chemical and thermal stability, high electric conductivity, tunable structures, and environmental friendliness without the leaching of the metals, compared to the metal or metal (hydr)oxides catalysts, such as a core–shell structured Fe_3_O_4_@C/CDs-Ag composite [9], N-Cu-doped biochar [10], a core–shell structured manganese oxide–carbon composite [11], and a composite of Fe and N-doped activated carbon [12]. The organic pollutant removal in the systems of CMs and PMS/PDS is featured by the involvement of non-radical pathways, i.e., electron transfer from a pollutant to the PMS/PDS, as well as the formation of non-radical reactive species such as singlet oxygen (^1^O_2_), while those of metal-based catalyst dominantly rely on the generation of radicals, such as sulfate (SO_4_^•−^) and hydroxyl (^−^OH) radicals (radical pathways) [2,4,6,13,14].

It has been demonstrated that the graphitic structures, which exist in most CMs, play a crucial role in the activation of PMS or PDS. It was reported that a higher degree of graphitization led to promoted PDS activation for the CMs with similar SSA [2,15] and improved the non-radical pathways, i.e., electron transfer, in the systems of CMs and PDS [14]. The structural defects, functional groups, and π electrons in the graphitic structures participate in the electron transfer processes between the materials, oxidants, i.e., PDS and PMS, and the pollutant, to facilitate the generation of radicals (SO_4_^•−^, ^−^OH, and O_2_^•−^) and non-radical reactive species (^1^O_2_), as well as to accelerate the electron withdrawal from a pollutant [3,6,9,13]. Therefore, it seems reasonable that a material with more graphitic structures would have a higher catalytic reactivity, and that the reactivity could be significantly enhanced when more graphitic structures are exposed, as in EG.

EG is prepared via the expansion of the distances of the layers of graphite [16]. The specific surface area of EG is generally much lower than other CMs [4], but the abundance of open surfaces can lead to faster contact with pollutants and oxidants, such as hydrogen peroxide (H_2_O_2_), PMS, and PDS, resulting in enhanced adsorption and/or oxidation on the surface. For example, an EG showed a much faster oil adsorption than other materials, such as polyimide/graphene aerogel, magnetic graphene/CNT foam, expanded perlite, and magnetic cobalt ferrite nanoparticles [16,17]. Moreover, it is thought that EG is ideal for investigating CMs as catalysts. EG is already highly graphitized that the role of the components in the graphitic structures would not be affected by non-graphitic structures [1,4,5]. In addition, mass transfer resistance in a catalytic system can be minimized because most of the surface of EGs is exposed [18].

However, contrary to the hypothesis, EG has demonstrated a poor catalytic activity, without modification using reactive materials such as CuFe_2_O_4_ [4], NiCo_2_O_4_ [19], Co [18], or gCN and nano-scale zero-valent iron [20]. Those modification procedures have intrinsic disadvantages, such as complex procedures and the generation of chemical wastewater. A simple thermal treatment would be one of the environmentally friendly and simple alternatives. It was reported in a limited number of literature sources that thermal treatment under inert gas conditions significantly changes the properties of graphitic structures and, therefore, the catalytic reactivity of CMs. The thermal treatment of apple tree-derived biochar showed an increase in C=O, basicity, defects, surface area, crystallinity, interlayer spacing of graphitic structure, and acetaminophen removal, activating PDS, while surface charge was significantly decreased [15]. Modification of ACs for enhanced dye adsorption generally involves thermal treatment [21]. Simple thermal treatment of a coal-based AC improved catalytic degradation of oxytetracycline in the presence of PDS [22]. In the systems of PDS and various biochars, 4-chlorophenol degradation was significantly improved when the biochars were annealed at 700~1100 °C [23]. Therefore, it seems clear that thermal treatment modulates pore structure, functionality, degree of graphitization, and the property of graphitic structure in CMs, affecting the catalytic reactivity [24].

In this regard, we investigated the changes in properties and catalytic degradation performance of TC by simple thermal modification of EG, which is a CM with a pure graphitic structure. The structure, pore property, and functional groups of EGs were investigated using XRD, N_2_ adsorption/desorption isotherm, FTIR spectroscopy, Raman spectroscopy, and XPS. TC degradation by EGs was evaluated by batch experiments in the presence of PDS. TC, along with chlortetracycline, oxytetracycline, lymecycline, doxycycline, and others, is a member of tetracyclines, a group of antibiotics inhibiting protein synthesis of microorganisms [25]. TC is one of the most widely used antibiotics for livestock, and up to 75% of the dosed TC is excreted [26]. Therefore, TC is frequently detected in water systems [27] and poses substantial threats to aquatic ecosystems, such as alteration of microbial community and increased antibiotic-resistant bacteria and genes [28].

## 2. Results and Discussion

### 2.1. TC Removal

EG was thermally annealed for two (2) h in a muffle furnace, at 350, 450, 550, and 650 °C, which are denoted as EG350, EG450, EG550, and EG650, respectively. The adsorption of TC onto EG was negligible (4.2%, 3.4 mg/g). It was improved by thermal treatment but was still poor (17.9~30.6%, 14.3~24.5 mg/g) (Figure S1). A low malachite green dye adsorption, ≤41.49 mg/g, by EG was also reported elsewhere [29].

**Figure 1:**
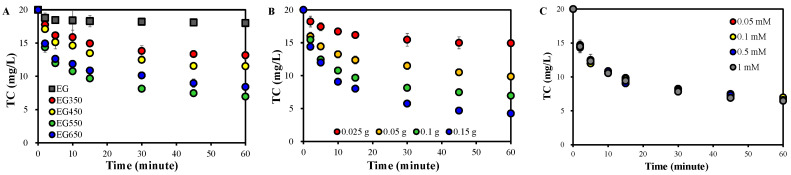
TC removal (**A**) by various EGs (EGs 0.1 g/L, PDS 0.1 mM), (**B**) at different EG550 doses in EG550+PDS (PDS 0.1 mM), and (**C**) at different PDS doses (EG550 0.1 g) in EG550+PDS (TC 20 mg/L).

However, TC removal was significantly enhanced in the presence of 0.1 mM PDS when thermally treated EGs were used (Figure 1A), suggesting a substantial role of the EGs in PDS activation. The TC removal rate was increased as thermal treatment temperature was increased from 350 °C to 550 °C, while it was decreased when the temperature was raised further to 650 °C.

The pseudo-first- and pseudo-second-order reaction kinetic models were poor fits to the experimental results (*r^2^* = 0.220~0.637). Therefore, the time courses of TC were fitted with the pseudo-first-order reaction model with general deactivation (Equations (1)–(3)) [30], providing excellent fits (*r^2^* = 0.945~0.974):

(1) $$\frac{dC_{t}}{dt}=-k_{1}C_{t}\left(\frac{a_{t}}{a_{0}}\right)$$

(2) $$\frac{a_{t}}{a_{0}}=\exp\left(-k_{D}t\right)$$

(3) $$\frac{dC_{t}}{dt}=-k_{1}C_{t}\cdot \exp\left(-k_{D}t\right)$$

where *C_t_* is OTC concentration (mg/L) at time *t* (min), *k* is reaction rate constant (min^−1^), *a*_0_ is initial activity, *a_t_* is the activity at time *t* (min), and *k_D_* is deactivation rate constant (min^−1^). The *k* was increased gradually from 0.84 × 10^−2^ min^−1^ (EG) to 8.70 × 10^−2^ min^−1^ (EG550) with increasing temperature and decreased to 5.02 × 10^−2^ min^−1^ (EG650), successfully describing the performance of the EGs (Figure S2).

Figure 1B displays that TC removal in EG550+PDS was improved with increasing EG550 dose, confirming the role of EG550 as a catalyst. However, the PDS dose showed no notable effect on TC removal in a range of 0.05~1.0 mM (Figure 1C), indicating that 0.05 mM PDS was more than the amount that was activated by 0.1 g EG550.

**Figure 2:**
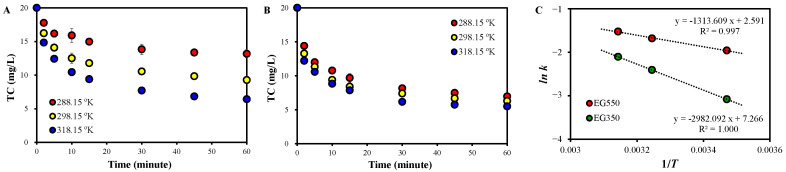
Effects of temperature on TC removal in (**A**) EG350+PDS and (**B**) EG550+PDS (EGs 0.1 g, PDS 0.1 mM, TC 20 mg/L), as well as (**C**) Arrhenius relationships.

The performance of EG+PDS was very poor, so no notable difference was found at different temperatures (Figure S3). On the other hand, TC removal was improved with increasing temperature for EG350+PDS and EG550+PDS (Figure 2A,B). The improvement was more pronounced in EG350+PDS than in EG550+PDS. The activation energy was calculated using the Arrhenius relationship. That in EG350+PDS and EG550+PDS was 27.2 and 10.9 kJ/mol, respectively (Figure 2C), suggesting that TC degradation was thermodynamically more favorable when EG550 was used than EG350.

### 2.2. Characteristics of EGs

It seems reasonable that the differences in the performance of the EGs were largely dependent on the different characteristics of the EGs because the structure and functional groups determine the reactivity of metal-free CMs [13,31]. Therefore, EG, EG350, and EG550 were characterized to find the key properties of improving PDS activation.

SEM images of the EGs are given in Figure S4. It shows that the microstructure of EG350 and EG550 was more wrinkled, twisted, and damaged than that of EG. This suggests that more exposed surfaces, pores, edges, and defects were formed by thermal treatment, which may be the reactive sites of PDS activation. Similar changes were also reported by a combination of gaseous ozonation and thermal treatment of an EG [32].

**Figure 3:**
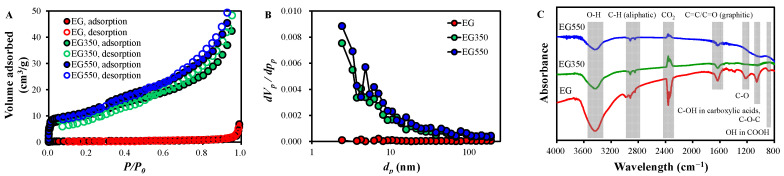
(**A**) N_2_ adsorption/desorption isotherm, (**B**) pore size distribution, and (**C**) FTIR spectra of EG, EG350, and EG550.

The results of N_2_ adsorption/desorption isotherm, SSA, total pore volume (*V_t_*), and average pore diameter (*d_a_*) of the EGs are presented in Figure 3A,B and Table 1. The N_2_ adsorption/desorption by EG was almost negligible. However, those of EG350 and EG550 showed type II isotherm with H3 hysteresis, suggesting that they are nonporous or microporous with the dominance of slit-like pores [33], as supported by the low SSA and *V_t_* (Table 1). On the other hand, it should be noted that EG550 showed slightly higher SSA, *V_t_*, and *d_a_* than EG350, and the pore size distributions of EG350 and EG550 were similar. However, the TC removal by EG550 was greatly superior to that of EG350. These strongly suggest that the difference in the catalytic reactivity between EG350 and EG550 was not attributable to their pore structure but to the differences in surface properties and structures [13,34].

FTIR spectrum of EG showed bands at 3600~3200, 2920~2800, ~1620, ~1210, ~1055, and ~880  cm^−1^, which are associated with O–H, aliphatic C–H, graphitic C=C/C=O, C-O, C–OH in carboxylic acids or C–O–C, and OH in COOH, respectively [5,35] (Figure 3C). The bands of C–O and C–O–C/C–OH disappeared for EG350 and EG550 via thermal decomposition [36]. The intensity of other bands was decreased as the temperature was increased from 350 °C to 550 °C, suggesting an increased graphitization degree [37].

**Figure 4:**
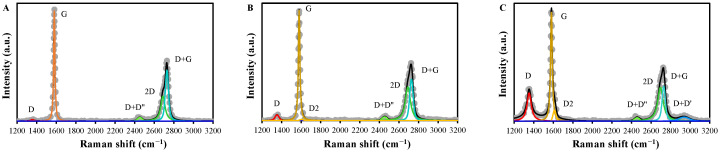
The Raman spectra of (**A**) EG, (**B**) EG350, and (**C**) EG550 (Grey symbols indicate raw data. Colored lines indicate deconvoluted bands).

The Raman spectra of the EGs are presented in Figure 4 and in Table 2. The spectrum of EG was deconvoluted into D, G, D+D’’, 2D, and D+G bands at 1357, 1580, 2453, 2689, and 2729 cm^−1^, respectively, which are assigned to *sp*^2^-C/*sp*^3^-C hybridization in defective structures (edge defects), *sp*^2^-C in ideal graphitic structures, an overtone, single graphene layer, and the splitting of 2D band, respectively [38,39,40]. Therefore, the ratio of the intensity of the D band to that of the G band (*I_D_/I_G_*) is indicative of the degree of defects, while that of the intensity of the 2D band to that of the G band (*I*_2*D*_*/I_G_*) is inversely proportional to the degree of stacking of graphitic layers [41].

The thermal treatment at 350 °C did not affect the abundance of the G band, suggesting that the ideal graphitic structures in the EG were not affected. However, the intensities of the D and the 2D band were increased notably, indicating an increase in defects, as indicated by the increase in *I_D_/I_G_* from 0.04 (EG) to 0.11 (EG350). It seems reasonable that the increased defects in EG350 were attributable to the detachment of stacked graphitic structures, which increases single-layer graphene-like structures, as demonstrated by the increase in *I*_2*D*_*/I_G_* from 0.75 (EG) to 0.98 (EG350). Increasing the temperature to 550 °C (EG550) resulted in a greater increase in defects and a slight increase in single-layer structures compared to those of EG350, as suggested by the higher *I_D_/I_G_* (0.66) and *I*_2*D*_*/I_G_* (1.01). This suggests that the degree of stacking in the EG was decreased under low temperatures, i.e., 350 °C, while the defects, related to *sp*^3^-C, hopping, vacancies, and on-site, were increased at 550 °C (EG550) [42]. The higher degree of defects of EG550 was also supported by the larger FWHM of the D band of EG550 than that of EG and EG350 [43]. On the other hand, the D2 band appeared only in EG350 and EG550 at 1606 and 1613 cm^−1^, respectively, indicating the formation of the disordered graphitic lattice, i.e., plane defects, by thermal treatment [39,44].

On the other hand, the Raman spectrum of EG650 showed a decrease in the intensity of D, D2, and 2D bands compared to that of EG550 (Figure S5), suggesting a decrease in defects and an increase in stacking degree [38,39,40,44]. These would be responsible for the reduced TC removal in EG650+PDS than in EG650+PDS (Figure 1A).

**Figure 5:**
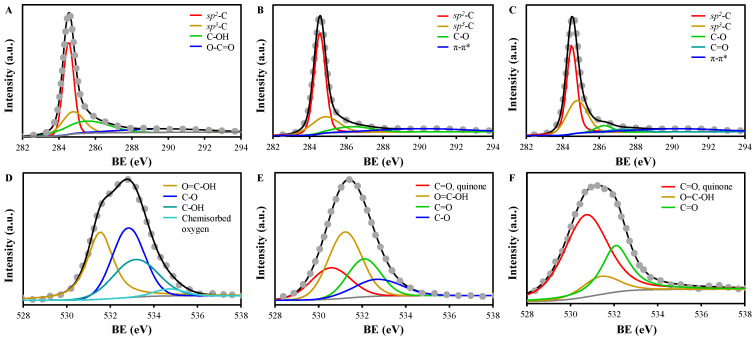
The C1s XPS spectra of (**A**) EG, (**B**) EG350, and (**C**) EG550, and the O1s XPS spectra of (**D**) EG, (**E**) EG350, and (**F**) EG550 (Grey symbols indicate raw data. Colored lines indicate deconvoluted peaks).

The XPS survey spectra showed the dominance of C and O in the EGs (Figure S6, Table S1). Based on the peak areas of C1s and O1s in XPS reports, the calculated content of C was 88.8%, 93.6%, and 96.0%, while that of O was 11.2%, 6.4%, and 4.0%, for EG, EG350, and EG550, respectively. High-resolution XPS spectra of C1s and O1s were deconvoluted for entire peak envelopes, considering similar and representative materials such as nuclear grade graphite and highly ordered pyrolytic graphite [45,46]. The results are given in Figure 5 and Table 3. C1s spectrum of EG showed peaks at 284.5, 284.8, 285.5, and 289.7 eV, corresponding to *sp*^2^-C, *sp*^3^-C, C–OH, C–O, and O–C=O, respectively, while O1s spectrum consisted of the peaks at 531.5, 532.8, 533.2, and 534.7 eV assigned to O=C–OH, C–O, C–OH, and O_ads_ [18,41,47]. Thermal treatment at over 350 °C resulted in the disappearance of C–OH, O–C=O, and O_ads_ in C1s and O1s spectra. Instead, C–O (286.3 eV) appeared in C1s of EG350, while C–O (286.3 eV) and C=O (287.3 eV) were found in C1s of EG550 [47]. New peaks were found in O1s spectra of EG350 and EG550, which are highly conjugated C=O groups such as quinone and pyrone (530.6~530.7 eV) and C=O (532.1 eV) [47,48]. The results demonstrated the thermal decomposition of hydrophilic groups, such as C–OH and O–C=O, under low temperatures [49]. In addition, the increase in graphitic carbons (*sp*^2^-C and *sp*^3^-C) and C=O in quinone suggest a higher degree of graphitization of EG350 and EG50 than EG, as also indicated by the results of Raman spectroscopy (Table 2 and Figure 4) [15,50]. Moreover, the sum of graphitic *sp*^2^-C and *sp*^3^-C was 64.1%, 77.4%, and 79.1%, while the ratio of *sp*^3^-C to *sp*^2^-C (*sp*^3^-C/*sp*^2^-C) was 0.50, 0.37, and 0.65, for EG, EG350, and EG550, respectively. This indicates that a low temperature annealing of EG increased graphitic structures (EG350), and defects were increased under a higher temperature (EG550).

EG650 was further investigated by XPS (Figure S7). Compared to EG550, the fraction of *sp*^3^-C was greatly increased, and that of C=O (quinone), one of the efficient PDS activation sites, was decreased. A considerable amount of O existed as chemically adsorbed. This suggests that the reactivity of EG560 is attributable not to O-containing groups but to high *sp*^3^-C content because high *sp*^3^-C/*sp*^2^-C is beneficial to PDS activation [51].

The results in Table 1, Table 2 and Table 3 and Figure 2, Figure 3, Figure 4 and Figure 5 suggest that the outstanding TC removal by EG550, compared to that by EG or EG350 (Figure 1A), was attributable to more developed graphitic structures, defects, *sp*^2^- and *sp*^3^- hybridized C, and basic groups (C=O), rather than more surfaces. The delocalized π electrons in Lewis basic sites and graphitic structures can break the O–O bonds in PDS to form SO_4_^−^ [3]. In addition, dangling bonds are formed at the defects in graphitic structures by the delocalized π electrons, which mediates and accelerates electron transfer from the structures to PDS [52,53]. The defects significantly affect the reactivity of graphitic materials because they violate the electronic homogeneity in ideal graphitic lattices, leading to the re-hybridization of σ and π orbitals and the changes in electron trajectories [54]. These promote both radical and non-radical pathways of organic pollutant degradation via accelerating the electron transfer to oxidants, such as PDS, to generate radicals, such as SO_4_^−^, OH^−^, and the electron abstraction from pollutants [6,13]. It is supported by the enhanced sulfamethoxazole degradation by PDS activated by multiwalled CNTs after graphitization at 2000 °C [9]. It is also supported by a report that the chemical reactivity of graphene monolayer was more than twice as high on edges as on planes [55].

### 2.3. Identification of Reactive Species in EG550+PDS

**Figure 6:**
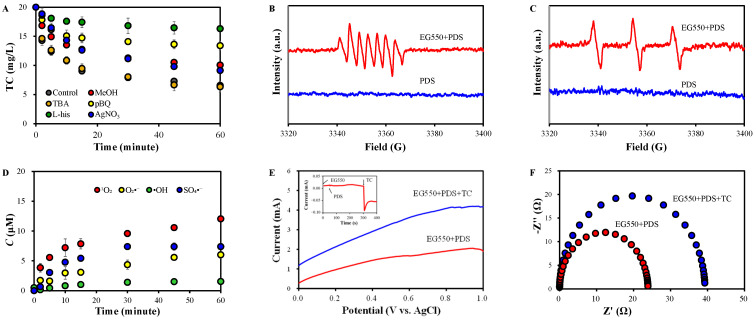
(**A**) TC removal in the presence of scavengers; ESR spectra using (**B**) DMPO (10 mM) and (**C**) TEMP (10 mM) (EG550 0.5 g/L, PDS 0.5 mM, TC 20 mg/L); (**D**) reactive species generation by probe chemical conversion; (**E**) LSV (inset is I-t curve) and (**F**) EIS of EG550+PDS and EG550+PDS+TC.

TC removal in EG550+PDS was investigated in the presence of scavengers (Figure 6A). TC removal was notably inhibited by MeOH but not by TBA. This suggests a significant and negligible involvement of SO_4_^•−^ and ^−^OH in EG550+PDS, respectively. MeOH, which has α-H, efficiently scavenges both SO_4_^•−^ and ^−^OH ($k_{SO_{4}^{•-}}$ = 1.6 × 10^7^ M^−1^s^−1^, kO•H = 1.9 × 10^9^ M^−1^s^−1^). On the other hand, TBA, which is a tertiary alcohol without an α-H, dominantly quenches ^−^OH (kO•H = (3.8~7.6) × 10^8^ M^−1^s^−1^) [56]. The generation of SO_4_^•−^ by activating PDS on CMs via delocalized π electrons and Lewis basic sites (C=O) has been reported (Equations (4) and (5)) [57].

The presence of pBQ and L-his greatly suppressed TC removal, indicating detrimental roles of both O_2_^•−^ and ^1^O_2_. In the systems containing PDS, O_2_^•−^ can be generated via various reactions of PDS on the defective sites of CMs and in bulk solutions, as well as from dissolved oxygen (Equations (6)–(8)) [4,58,59]. However, it is thought that the inhibition by pBQ is attributable to the reduced ^1^O_2_ formation from O_2_^•−^, rather than a decreased attack on TC by O_2_^•−^. O_2_^•−^ has a negative reduction potential of −0.81 eV [60]. However, O_2_^•−^ is one of the most important precursors of ^1^O_2_ in an aqueous environment (Equations (9) and (10)) [56]. The inhibition by quenching ^1^O_2_ was superior to that by O_2_^•−^, indicating that the generation of ^1^O_2_ was mediated not only by O_2_^•−^ but also by other pathways. It was reported that ^1^O_2_ is generated by the oxidation of epoxy structures, which are formed via the oxidation of C=O in defects in CMs (Equations (11) and (12)) [61]. In graphitic structures, electron-poor graphitic N and C atoms near the graphitic N were also proposed as the sites of PDS activation [62]. However, they can be excluded in EG550+PDS because of the negligible N content [63].

It should also be noted that TC removal was significantly inhibited by AgNO_3_, a common electron quencher [64], suggesting a substantial involvement of electron transfer.

(4) $$S{O_{4}}^{2-}+C=C=O\to S{O_{4}}^{•-}+C=C=O^{+}$$

(5) $$S{O_{4}}^{2-}+C-\pi \to S{O_{4}}^{•-}+C-\pi^{+}$$

(6) $$O_{2}+e^{-}\left(defects\right)\to {O_{2}}^{•-}$$

(7) $$2S_{2}{O_{8}}^{2-}+2H_{2}O+e^{-}(defects)\to 4S{O_{4}}^{2-}+{O_{2}}^{•-}+4H^{+}$$

(8) $$S_{2}{O_{8}}^{2-}+H{O_{2}}^{-}\to S{O_{4}}^{-}+S{O_{4}}^{2-}+H^{+}+{O_{2}}^{•-}$$

(9) 2O2•−+2H2O→H2O2+2OH−+O21

(10) 2O2•−+2H+→H2O2+O21

(11) $$R-C=O+S_{2}{O_{8}}^{2-}+2OH^{-}\to R-C\begin{array}{c} /\\ \backslash \end{array}\begin{array}{c} O\\ |\\ O \end{array}+2S{O_{4}}^{2-}+H_{2}O$$

(12) R−C/\O|O+S2O82−+2OH−→R−C=O+2SO42−+H2O+O21

The ESR spectra of EG550+PDS showed clear signals of 5,5-dimethylpyrrolidone-2-(oxy)-(1) (DMPO-X) and TEMP-^1^O_2_ [14,65] (Figure 6B,C), supporting the involvement of SO_4_^−^ and ^1^O_2_ in TC degradation.

The concentrations of reactive species were investigated using the conversion of chemical probes in the EG550+PDS system [6]. The probes used were HBA, SA, NBT, and DPBF, which selectively react with SO_4_^•−^, ^−^OH, O_2_^•−^, and ^1^O_2_, respectively. Results showed that the generated amount was in the order of ^1^O_2_ > O_2_^•−^ > SO_4_^•−^ > ^−^OH (negligible), confirming the significant role of ^1^O_2_ and O_2_^•−^ as the precursors of ^1^O_2_ (Figure 6C). It can be considered that the results of a quenching experiment and ESR spectroscopy indicate that the contribution of SO_4_^−^ is not dominant in this system (Figure 6A,B). However, it seems reasonable that the results in Figure 6C also support the contribution of SO_4_^•−^, considering a higher redox potential of SO_4_^•−^ (2.5~3.1 V) compared to that of ^1^O_2_ (1.07 V) [65].

The contributions of reactive species were quantified using a kinetic method. The TC removal rate constants in the presence of the scavengers (Figure S8) were used, and it was assumed that the reactions between TC and reactive species are parallel (Text S1) [66]. The contribution of O_2_^•−^ was not considered because it has a negative redox potential and mainly serves as the precursor of ^1^O_2_ [56]. The contribution of SO_4_^•−^, ^−^OH, ^1^O_2_, and electron transfer was 21.7%, 1.1%, 42.6%, and 34.6%, respectively.

The negligible involvement of OH was demonstrated by the results of quenching experiments (Figure 6A), the absence of DMPO-OH signals (Figure 6B), the low ^−^OH generation (Figure 6C), and the calculated contribution. This suggests that the propagation of SO_4_^•−^ to ^−^OH did not occur, probably because of the instantaneous consumption of SO_4_^•−^ upon its generation [62].

Figure 6E shows significantly higher currents in EG550+PDS+TC than in EG550+PDS, indicating a substantial electron transfer from TC, an electron donor, to PDS, an electron acceptor [67], as also demonstrated by the inhibition of TC removal by an electron scavenger (AgNO_3_) (Figure 6A). The inset of Figure 6E shows a fluctuation in the current when PDS was introduced to a reactor, which contains EG550 and distilled, deionized water. This indicates the formation of meta-stable activated PDS bound on the surface of EG550 (EG550-PDS *) [67] and the involvement of radical pathways [14]. On the other hand, the current changed significantly when TC was introduced [14], suggesting the mediation of electron transfer by EG550 from TC to PDS. More, the radius of the Nyquist semicircle in EG550+PDS+TC was smaller than in EG550+PDS (Figure 6F), suggesting faster electron transfer in the presence of both PDS and TC. Based on the results in Figure 6, it seems clear that both radical and non-radical pathways are involved in EG550+PDS+TC.

### 2.4. TC Degradation Intermediates and Pathways

**Figure 7:**
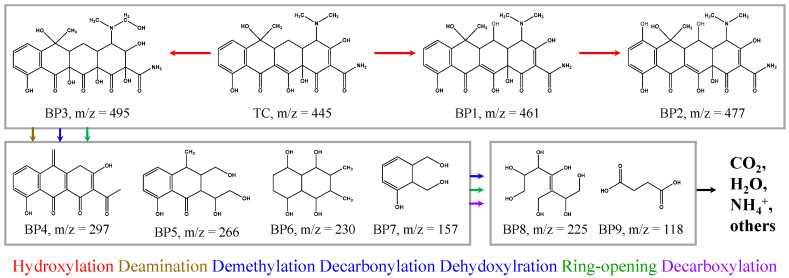
Proposed TC degradation pathways in EG550+PDS.

The detected intermediates of TC degradation in EG550+PDS and HPLC-MS/MS chromatograms are provided in Table S2 and Figure S9, respectively. Nine (9) byproducts were found with *m/z* values of 495, 461, 477, 297, 266, 230, 225, 157, and 118. The proposed pathways are illustrated in Figure 7. It was shown that TC molecules were subjected to hydroxylation and subsequent deamination, demethylation, dehydroxylration, decarbonylation, and ring-opening [68].

At first, TC was hydroxylated, which is known as one of the major steps of TC degradation, as previously reported [69,70], to form BPs 1, 2, and 3. The BP1 was generated by the hydroxylation at the C5 position, and it was further hydroxylated at C7–C8 to BP2. TC was hydroxylated via another pathway at electron-donating moieties, i.e., C2–C3, C11–C12, and methyl group, to generate BP3. The hydroxylated BPs underwent deamination, demethylation, dehydroxylation, decarbonylation, and ring-opening at various degrees to form BPs 4~7. The amine groups, aromatic rings, and phenolic hydroxyl groups are of high electron density and easily attacked by reactive species [69]. It is speculated that the ring-opening would start from the cleavage of C12a–C1 via the decarbonylation at the enolic acetylacetone moiety (C12a–C4), which has relatively lower bond energy [70]. They were more degraded by decarboxylation as well as further demethylation, dehydration, decarbonylation, and ring-opening to the BPs 8 and 9, and, finally, to CO_2_, H_2_O, NH_4_^+^, and other lower molecules.

### 2.5. Effects of Co-Existing Ions

**Figure 8:**
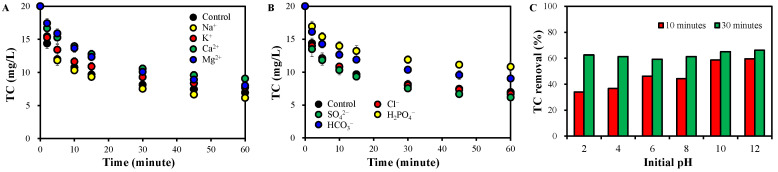
TC removal in the presence of (**A**) cations and (**B**) anions, as well as (**C**) at different initial pH (EG550 0.1 g, PDS 0.1 mM, cations, and anions 10 mM).

The effects of cations and anions, commonly found in surface water, groundwater, and secondary treatment effluent, were investigated to evaluate the feasibility of practical applications (Figure 8).

TC removal was not notably affected by monovalent cations (Na^+^ and K^+^) but was suppressed substantially by divalent cations (Ca^2+^ and Mg^2+^) (Figure 8A). The TC removal rate constant (*k* in Equation (3)) was 0.1151 ± 0.0081 min^−1^ in the control and in the presence of monovalent cations, while it was 0.0553 and 0.0496 min^−1^ when Ca^2+^ and Mg^2+^ were introduced, respectively (Figure S10). Monovalent cations rarely participate in PDS activation because of their high chemical stability and low Lewis acidity [71]. However, the role of divalent cations is diverse [9]. They can enhance the catalytic degradation by bridging negatively charged organic pollutants, CMs surfaces, and PDS to enhance electron transfer between them [9]. O-containing groups in CMs promote the binding by increasing negative charges via dissolution in the aqueous phase [9]. However, they can reduce available surface reactive sites of CMs via the aggregation of CMs by the neutralization of surface charges [9,20]. The higher Lewis acidity of Ca^2+^ and Mg^2+^ would affect the PDS/PMS activation both positively and negatively. Lewis acidic non-redox metals can promote PDS/PMS decomposition, but this can lead to O_2_ formation, reducing the generation of reactive species [71]. Therefore, it is suggested that the inhibition by aggregation and disproportionation was stronger than the promotion by the bridging, in EG550+PDS with Ca^2+^ or Mg^2+^.

Cl^−^ and SO_4_^2−^ did not notably affect the TC removal in EG550+PDS, while it was significantly reduced by H_2_PO_4_^−^ and HCO_3_^−^ (Figure 8B). The slight effects of Cl^−^ and SO_4_^2−^ are attributable to their reactions in a system activating PDS. It is known that SO_4_^•−^ was scavenged by Cl^−^ to generate Cl^−^ and SO_4_^2•−^. The redox potential of Cl^−^ (2.4~2.5 V) is comparable to that of SO_4_^•−^ (1.8~2.7 V) and is higher than that of ^1^O_2_ (1.07 V) [65]. In addition, the reaction is reversible with similar rate constants for forward and backward reactions (Equation (13)) [72]. Therefore, the influence of Cl^−^ would not be significant. On the other hand, SO_4_^2−^ does not react with PDS or change the solution pH [73]. Excess SO_4_^2−^ reacts with SO_4_^•−^ to form PDS anion, donating electrons (Equation (14)). However, the regenerated PDS can further be activated, so SO_4_^2−^ does not exert a notable effect on PDS activation.

It should be noted that no reaction between ^1^O_2_ with Cl^−^/SO_4_^2−^ has been reported to date [65]. Assuming no interaction, the slight effects of Cl^−^/SO_4_^2−^ can be assigned to the reactions of SO_4_^•−^ with Cl^−^/SO_4_^2−^, considering negligible ^−^OH formation and the dominant role of ^1^O_2_.

The inhibition by H_2_PO_4_^−^ and HCO_3_^−^ is attributable to the scavenging of SO_4_^•−^, which was identified as one of the major reactive species in EG550+PDS (Figure 6A,B), to form H_2_PO_4_^−^ and HCO_3_^−^ (Equations (15)–(17)) [73,74]. The redox potential of H_2_PO_4_^−^ is as high as that of SO_4_^•−^ (2.65 V); however, the organic pollutants degradation rate of H_2_PO_4_^−^ is significantly lower than that of SO_4_^•−^ [73]. HCO_3_^−^ has a lower potential of 1.65 V [73]. A reactive anion, i.e., percarbonates (HCO_4_^−^) is formed via the interaction between HCO_3_^−^ and SO_4_^•−^ (Equation (19)); however, its organic pollutant degradation is very slow [75]. Additionally, the inhibition by H_2_PO_4_^−^ and HCO_3_^−^ is also attributable to the competition of them with negatively charged PDS and TC for the reactive sites on EG550 [73].

However, it should be noted that the performance of EG550+PDS would be more stable than the systems using other CMs because the inhibition in Figure 8A,B was not as significant as in other systems [9,21].

(13) $$\begin{array}{l} S{O_{4}}^{•-}+{Cl}^{-}\overset{forward}{\underset{backward}{⇄}}\to S{O_{4}}^{2-}+Cl^{•}\\ k_{forward}=2.47-6.6\times 10^{8}M^{-1}s^{-1},\;k_{backward}=2.5\times 10^{8}M^{-1}s^{-1} \end{array}$$

(14) $$S{O_{4}}^{2-}+S{O_{4}}^{•-}\to S_{2}{O_{8}}^{2-}+e^{-}$$

(15) $$S{O_{4}}^{•-}+H_{2}PO_{4}^{-}\to S{O_{4}}^{2-}+H_{2}PO_{4}^{•}$$

(16) $$S{O_{4}}^{•-}+HCO_{3}^{-}\to S{O_{4}}^{2-}+CO_{3}^{•}+H^{+}$$

(17) $$S{O_{4}}^{•-}+HCO_{3}^{-}\to S{O_{4}}^{2-}+HCO_{3}^{•}$$

(18) 2O2•−+2H+→H2O2+O21

(19) $$HCO_{3}^{-}+S_{2}{O_{8}}^{2-}+2OH^{-}\to HCO_{4}^{-}+2S{O_{4}}^{2-}+H_{2}O$$

The effects of pH on TC removal are provided in Figure 8C. TC removal was improved as pH increased. Under acidic conditions, the major reactive species is SO_4_^•−^, generated via acid catalysis (Equations (20) and (21)) [76]. However, O-group in HS_2_O_8_^−^ can form H-bonds with positive charges (H^+^), resulting in stabilization [77]. In addition, the abundance of SO_4_^−^ leads to more self-scavenging of SO_4_^•−^ (Equation (22)). Under all pH conditions and alkaline conditions, ^−^OH can be formed via reaction with H_2_O and OH^−^, respectively (Equations (23) and (24)) [78]. Under stronger alkaline conditions, SO_4_^•−^ and O_2_^•−^ are generated via HO_2_^−^, formed from S_2_O_8_^2−^ hydrolysis (Equations (25) and (26)) [79]. Then, more ^1^O_2_ can be generated from the O_2_^•−^ (Equations (9) and (10)). Meanwhile, pH did not change notably in the reaction period, except when the initial pH was 6 and 8 (Figure S11). The pH decreased when the initial pH was near neutral, i.e., 6 and 8, by the H^+^ supply from PDS hydrolysis [52].

(20) $$S_{2}{O_{8}}^{2-}+H^{+}\to HS_{2}O_{8}^{-}$$

(21) $$HS_{2}O_{8}^{-}\to S{O_{4}}^{•-}+S{O_{4}}^{2-}+H^{+}$$

(22) $$S{O_{4}}^{•-}+S{O_{4}}^{•-}\to S_{2}{O_{8}}^{2-}$$

(23) SO4•−+H2O→SO42−+O•H+H+

(24) SO4•−+OH−→SO42−+O•H

(25) $$S_{2}{O_{8}}^{2-}+2H_{2}O\to HO_{2}^{-}+2S{O_{4}}^{2-}+H^{+}$$

(26) $$S_{2}{O_{8}}^{2-}+HO_{2}^{-}\to S{O_{4}}^{•-}+S{O_{4}}^{2-}+{O_{2}}^{•-}+H^{+}$$

## 3. Materials and Methods

### 3.1. Materials

TEMP (C_9_H_19_N, ≥99%), DMPO (C_6_H_11_NO, ≥97%), hydrochloric acid (HCl, 37%), MeOH (CH_3_OH, ≥99.9%), orthophosphoric acid (H_3_PO_4_, ≥99.9%), pBQ (C_6_H_4_(=O)_2_, ≥98%), potassium bromide (KBr, ≥99%), potassium chloride (KCl, 99.0–100.5%), potassium peroxydisulfate (K_2_S_2_O_8_), sodium hydroxide (NaOH, ≥97%), TBA (CH_3_)_3_COH, ≥99%), and TC hydrochloride (C_22_H_24_N_2_O_8_·HCl) were purchased from Merck KGaA (Darmstadt, Germany). All reagents were of analytical grade and were used as received. EG was procured from Samjung C&G Co., Ltd. (Pyungtask, Republic of Korea). The Aquapuri 551 system (Youngin Chromass, Anyang, Republic of Korea) was used to prepare distilled, deionized water. The EG was put in quartz boats and was dried at 150 °C for twelve (12) h in a muffle furnace (SH-FU-27MG, Samheung Co., Ltd., Seoul, Republic of Korea). Then the temperature was elevated at a heating rate of 5 °C/min. The temperature was maintained for two (2) h at 350, 450, 550, or 650 °C before it was naturally cooled to room temperature. The EGs were denoted as EGX where X is the thermal treatment temperature. The atmosphere in the furnace was not controlled and did not have any input of an inert gas.

### 3.2. Characterization

XRD patterns were recorded with a DB Advance X-ray diffractometer (Bruker, Billerica, MA, USA) at a scanning speed of 0.2°/min in the *2θ*s of 3°~89.14°. The pore size distribution and specific surface area (SSA) were analyzed by N_2_ adsorption and desorption at 77 °K using the Brunauer–Emmett–Teller (BET) and the Barrett–Joyner–Halenda (BJH) isotherms with BELSORP-max (Microtrac, Osaka, Japan). Spectrum One System (Perkin-Elmer, Waltham, MA, USA) was used to obtain FTIR spectra of the pellets prepared from the mixtures of 0.2 mg sample and 200 mg KBr, in the wavelengths of 4000–400 cm^−1^. Raman spectra at Raman shifts of 1200–3400 cm^−1^ were obtained with an inVia Raman microspectrometer (Renishaw, UK). XPS analyses were conducted using K-Alpha XPS (Thermo Electron, Waltham, MA, USA) with a monochromatic Al K-Alpha radiation source. It was also used to obtain high-resolution XPS spectra of C1s and O1s with a pass energy of 30 eV in 0.1 eV steps. Electron spin resonance (ESR) spectra were recorded using JES-TE300 (Jeol, Tokyo, Japan, X-band). The center field was 3389.9 G, sweep width was 100.0 G, power was 2.5 mW, modulation frequency was 100 kHz, and sweeping time was 2 min. DMPO and TEMP were used to trap reactive species.

### 3.3. TC Removal

Batch experiments were performed in 250 mL amber round-bottom flasks at room temperature. The EGs were dispersed in a certain amount of DIW via sonication, followed by the injection of the aqueous stock solutions of TC (500 mg/L) and PDS (10 mM). The concentration of EGs, PDS, and TC was 0.1 g/L, 0.1 mM, and 20 mg/L, respectively, unless noted otherwise. The mixture was continuously stirred. Aliquots were taken at predetermined times and were filtered using 0.45 μm PVDF membranes for analysis. The effects of radical scavengers on the TC removal in EG/PDS were investigated via batch experiments in the presence of scavenger chemicals. MeOH, TBA, pBQ, and L-his were used to scavenge OH and SO_4_^−^, OH, O_2_^−^, and ^1^O_2_ [56,60].

### 3.4. Analysis

TC concentration was measured using a high-performance liquid chromatography (HPLC) system (YL9100 Plus, Youlngin, Republic of Korea). The mobile phase was composed of 0.01 M oxalic acid, acetonitrile, and methanol (70:20:10, *v/v/v*), and a C18 column (Eclipse Plus, Agilent, CA, USA) was used. The flowrate, temperature, detection wavelength, and injection volume were 1 mL/min, 30 °C, 360 nm, and 25 μL, respectively. The intermediates of TC degradation in the system of EG550 and PDS (EG550+PDS) were investigated by a liquid chromatography–tandem mass spectrometry (LC-MS/MS), using Agilent 6460 triple quad mass spectrometer (Agilent Technologies, Santa Clara, CA, USA), equipped with ZORBAX Eclipse plus C18 column (2.1 mm, 3.5 μm). The column temperature and injection volume were 30 °C and 5 μL, respectively. The total flowrate of the mobile phase was 1.0 mL/min, with gradient elution of 0.1% formic acid in H_2_O and 0.1% formic acid in acetonitrile.

## 4. Conclusions

This study investigates the changes in characteristics and catalytic performance of EG by simple thermal treatment to investigate the effects of the property of core reactive structure of CMs, i.e., graphitic structures, on their catalytic reactivity.

In the presence of PDS, TC removal by pristine EG was poor but was greatly improved by thermal treatment at 350~650 °C. EG modified at 550 °C (EG550) showed the best performance. The TC removal rate constant of EG550+PDS was 10.3 times that of EG+PDS at 0.1 g/L EGs, 0.1 mM PDS, and 20 mg/L TC.

Thermal modification of EG increased SSA (40.2 m^2^/g for EG550), though it was lower than that of other CMs, such as AC. The surfaces of EG550 were rougher and more damaged than those in EG and EG350. The results of FTIR spectroscopy, Raman spectroscopy, and XPS showed that thermal treatment of EG resulted in increased graphitization degree, C content, degree of defects, and highly conjugated C=O (quinone), as well as decreased stacking degree, O content, C-OH, C-O-C, O-C=O, and O_ads_. The involvement of radical and non-radical pathways, such as SO_4_^−^ and O_2_^−^, ^1^O_2_, and electron transfer, was demonstrated in EG550+PDS, which led to TC degradation via hydroxylation, demethylation, dehydroxylation, decarbonylation, and ring-opening. TC removal in EG550+PDS was not significantly affected by common ions in water systems, except for H_2_PO_4_^−^ and HCO_3_^−^.

The results in this study strongly suggest that the reactivity of CMs is largely determined by the characteristics of graphitic structure, especially reactive sites such as defects and functional groups, rather than by the abundance of graphitic structure or surfaces. It is believed that this study provides valuable information about the design and application of CMs in catalytic degradation of refractory organic pollutants in water.

## Figures and Tables

**Table 1:** Results of N_2_ adsorption/desorption isotherm.

	SSA (m^2^/g)	*V_t_* (cm^3^/g)	*d_a_* (nm)
EG	1.209	0.0092	30.5
EG350	34.942	0.1315	15.1
EG550	40.219	0.1556	15.5

**Table 2:** Results of Raman spectroscopy.

		D	G	D2	D+D’’	2D	D+G	D+D’	*I_D_/I_G_*	*I*_2*D*_ */I_G_*
EG	Center (cm^−1^)	1357	1580		2453	2689	2729		0.04	0.75
	Area (%)	1.4	35.4		3.1	26.4	33.8			
	FWHM (cm^−1^)	43.3	15.9		53.9	52.1	38.5			
EG	Center (cm^−1^)	1353	1580	1606	2455	2692	2728		0.11	0.98
350	Area (%)	3.8	34.9	0.6	2.4	34.1	24.2			
	FWHM (cm^−1^)	47.5	1.0	41.3	57.0	62.4	43.0			
EG	Center (cm^−1^)	1353	1581	1613	2458	2696	2730	2938	0.66	1.01
550	Area (%)	18.4	28.0	3.4	1.9	28.3	14.8	5.2		
	FWHM (cm^−1^)	53.0	20.9	36.4	59.3	67.7	39.6	125.8		
EG	Center (cm^−1^)	1353	1579	1612	2462	2679	2719			
650	Area (%)	1.3	34.8	0.8	1.4	16.0	45.7		0.04	0.46
	FWHM (cm^−1^)	23.9	23.6	17.4	46.7	100.1	41.7			

**Table 3:** Results of XPS.

		C1s							O1s					
		*sp*^2^-C	*sp*^3^-C	C-OH	C-O	C=O	O-C=O	π-π* shake-up	C=O (quinone)	O=C-OH	C=O	C-O	C-OH	O_ads_ ^a^
EG	BE (eV)	284.5	284.8	285.5			289.7			531.5		532.8	533.2	534.7
	Fraction (%)	42.6	21.5	22.9			13.0			36.1		35.0	25.7	3.2
	FWHM (eV)	0.69	1.42	2.86			5.68			1.42		1.75	2.40	1.60
EG	BE (eV)	284.5	284.8		286.3			290.1	530.6	531.2	532.1	532.7		
350	Fraction (%)	56.4	21.0		10.2			12.4	22.4	40.6	22.5	14.5		
	FWHM (eV)	0.73	1.82		2.55			5.50	2.13	1.82	1.80	2.59		
EG	BE (eV)	284.5	284.8		286.3	287.3		290.2	530.7	531.4	532.1			
550	Fraction (%)	48.0	31.2		5.2	3.9		11.8	62.6	10.8	26.6			
	FWHM (eV)	0.67	1.34		1.10	1.94		5.35	2.37	1.98	1.54			
EG	BE (eV)	284.3	284.8	285.3		287.5		290.7	530.1	531.5				533.6
650	Fraction (%)	1.8	61.6	25.5		4.5		6.7	31.8	37.7				30.5
	FWHM (eV)	0.60	0.74	2.26		2.23		3.56	1.94	2.09				4.85

Note. ^a^. chemi-sorbed oxygen.

## Data Availability

The original contributions presented in this study are included in the article/Supplementary Materials.

## Supplementary Materials

The following supporting information can be downloaded at: https://www.mdpi.com/article/10.3390/molecules30112275/s1, Text S1: Quantitation of the contribution of reactive species by kinetic method. Figure S1: TC removal by adsorption onto EGs; Figure S2: TC removal rate constants of EGs; Figure S3: Effects of temperature on TC removal in EG+PDS; Figure S4: SEM images of (A) EG, (B) EG350, and (C) EG550; Figure S5: Raman spectrum of EG650; Figure S6: The XPS survey spectra of EG, EG350, and EG550; Figure S7: (A) Survey spectrum and high resolution spectra of (B) C1s and (C) O1s of EG650; Figure S8: TC removal rate constant (k) in Control and in the presence of scavengers; Figure S9: HPLC-MS/MS spectra of (A) TC and (B)~(D) the TC degradation intermediates in EG550+PDS at 60 minutes of reaction time; Figure S10: TC removal rate constant (k) in Control and in the presence of 10 mM cations; Figure S11: Changes in pH in EG550+PDS at different initial pH. Table S1: The components of XPS survey spectra. Table S2: Degradation products of tetracycline.

## Abbreviations

AC	activated carbon
CM	carbonaceous material
CNT	carbon nanotube
DMPO	5,5-dimethyl-1-pyrroline *n*-oxide
DPBF	1,3-Diphenylisobenzofuran
EG	expanded graphite
ESR	electron spin resonance
FTIR	Fourier transform infrared
FWHM	full width at half maximum
gCN	graphitic carbon nitride
HBA	*p*-hydroxybenzoic acid
HPLC	high-performance liquid chromatography
L-his	L-histidine
MeOH	methyl alcohol
NBT	nitrotetrazolium blue chloride
O_ads_	adsorbed oxygen
pBQ	*p*-benzoquinone
PDS	peroxydisulfate
PMS	peroxymonosulfate
PVDF	polyvinylidene fluoride
SA	salicylic acid
SEM	scanning electron microscopy
SSA	specific surface
TBA	*t*-butanol
TC	tetracycline
TEMP	2,2,6,6-tetramethylpiperidine
XPS	X-ray photoelectron spectroscopy
XRD	X-ray diffraction

## References

[1] Lee J., von Gunten U., Kim J.-H. (2020). Persulfate-Based Advanced Oxidation: Critical Assessment of Opportunities and Roadblocks. Environ. Sci. Technol..

[2] Yang Y., Cui Y., Zhao K., Sun H., Zhang W., Kuang P., Ma X., Zhu K., Ma K. (2025). Unleashing the Potential of Biomass-Doped Sludge Biochar: Promotion of Persulfate Activation by Biochar-Derived Dissolved Organic Matter. Sep. Purif. Technol..

[3] Chen X., Oh W.-D., Lim T.-T. (2018). Graphene- and CNTs-Based Carbocatalysts in Persulfates Activation: Material Design and Catalytic Mechanisms. Chem. Eng. J..

[4] Liu S., Lai C., Zhou X., Zhang C., Chen L., Yan H., Qin L., Huang D., Ye H., Chen W. (2022). Peroxydisulfate Activation by Sulfur-Doped Ordered Mesoporous Carbon: Insight into the Intrinsic Relationship between Defects and ^1^O_2_ Generation. Water Res..

[5] Hu Y., Chen D., Zhang R., Ding Y., Ren Z., Fu M., Cao X., Zeng G. (2021). Singlet Oxygen-Dominated Activation of Peroxymonosulfate by Passion Fruit Shell Derived Biochar for Catalytic Degradation of Tetracycline through a Non-Radical Oxidation Pathway. J. Hazard. Mater..

[6] Nguyen T.T., Kim D.G., Ko S.O. (2024). Catalytic Degradation of Acetaminophen by C and O Co-Doped Graphitic Carbon Nitride: Peroxymonosulfate vs. Peroxydisulfate. Chem. Eng. J..

[7] Reza M.S., Afroze S., Kuterbekov K., Kabyshev A., Bekmyrza K.Z., Haque M.N., Islam S.N., Hossain M.A., Hassan M., Roy H. (2023). Advanced Applications of Carbonaceous Materials in Sustainable Water Treatment, Energy Storage, and CO2 Capture: A Comprehensive Review. Sustainability.

[8] Kurniawan T.A., Ali S., Mohyuddin A., Haider A., Riaz M., Khan S., Othman M.H.D., Goh H.H., Anouzla A., Aziz F. (2024). Cultivating Sustainability: Harnessing Biochar-Derived Composites for Carbon-Neutral Wastewater Treatment. Process Saf. Environ. Prot..

[9] Zhang H.-C., Han J.-J., Zhang X., Guo P.-C., Xie D.-H., Sheng G.-P. (2021). Undiscovered Multiple Roles of Multivalent Cations in the Pollutant Removal from Actual Water by Persulfate Activated by Carbon Materials. ACS EST Eng..

[10] Zhong Q., Lin Q., Huang R., Fu H., Zhang X., Luo H., Xiao R. (2020). Oxidative Degradation of Tetracycline Using Persulfate Activated by N and Cu Codoped Biochar. Chem. Eng. J..

[11] Liu L., Liu Z., Chen Y., Zhou X., Kong W., Lv L., Xu Q., Gao B., Li Q. (2021). In-Situ Synthesis of Manganese Oxide-carbon Nanocomposite and Its Application in Activating Persulfate for Bisphenol F Degradation. Sci. Total Environ..

[12] Cui J., Zhao Z., Ren Y., Chen B., Rheinlander C., Moore A.X., Li L. (2024). Excellent Cycle Stability of Fe Loaded on N-Doped Activated Carbon for Microwave Hydrogenolysis of Lignin. Chem. Eng. J..

[13] Zhu K., Shen Y., Hou J., Gao J., He D., Huang J., He H., Lei L., Chen W. (2021). One-Step Synthesis of Nitrogen and Sulfur Co-Doped Mesoporous Graphite-like Carbon Nanosheets as a Bifunctional Material for Tetracycline Removal via Adsorption and Catalytic Degradation Processes: Performance and Mechanism. Chem. Eng. J..

[14] Liang Y., Cui J., Ning C., Zhang F., Liang F., Gao J. (2025). Construction of Adsorption-Oxidation Bifunction-Oriented Sludge Biochar for Non-Radical Ofloxacin Degradation via Persulfate: Emphasizing the Important Role of N-Species and Graphitized Structure. Sep. Purif. Technol..

[15] Kim D.-G., Ko S.-O. (2020). Effects of Thermal Modification of a Biochar on Persulfate Activation and Mechanisms of Catalytic Degradation of a Pharmaceutical. Chem. Eng. J..

[16] Elbidi M., Resul M.F.M.G., Rashid S.A., Salleh M.A.M. (2023). Preparation of Eco-Friendly Mesoporous Expanded Graphite for Oil Sorption. J. Porous Mater..

[17] Elbidi M., Salleh M.A.M., Rashid S.A., Resul M.F.M.G. (2024). The Potential of Thermally Expanded Graphite in Oil Sorption Applications. RSC Adv..

[18] Fang X., Feng Y., Li X., Ding D., Wang X., Zhang D. (2024). Efficient Fenton-like Catalysis Enabled by Single Cobalt Atoms Anchored on Expanded Graphite: Remarkable Intrinsic Activity of Co-N4 Sites and the Enhanced Mass Transfer Facilitated by Gradient Mesopore Structure. Chem. Eng. J..

[19] Xu M., Zhou H., Wu Z., Li N., Xiong Z., Yao G., Lai B. (2020). Efficient Degradation of Sulfamethoxazole by NiCo_2_O_4_ Modified Expanded Graphite Activated Peroxymonosulfate: Characterization, Mechanism and Degradation Intermediates. J. Hazard. Mater..

[20] Wang Y., Zhang W., Shang J., Shen C., Joseph S.D. (2019). Chemical Aging Changed Aggregation Kinetics and Transport of Biochar Colloids. Environ. Sci. Technol..

[21] Azam K., Shezad N., Shafiq I., Akhter P., Akhtar F., Jamil F., Shafique S., Park Y.-K., Hussain M. (2022). A Review on Activated Carbon Modifications for the Treatment of Wastewater Containing Anionic Dyes. Chemosphere.

[22] Kim D.-G., Kim T.-H., Ko S.-O. (2022). Enhanced Catalytic Activity of a Coal-Based Powdered Activated Carbon by Thermal Treatment. Water.

[23] Suh S.I., Woo H., Song S.-Y., Park D., Ahn Y.-Y., Kim E., Lee H., Kim D.-W., Lee C., Ok Y.S. (2023). Comparative Assessment of Biochars from Multiple Sources Based on Persulfate Activation Capability: Role of Iron Component in Enhancing Thermal Treatment Effect on Carbocatalytic Performance. Appl. Catal. B Environ..

[24] Crincoli K.R., Jones P.K., Huling S.G. (2020). Fenton-Driven Oxidation of Contaminant-Spent Granular Activated Carbon (GAC): GAC Selection and Implications. Sci. Total Environ..

[25] Scaria J., Anupama K.V., Nidheesh P.V. (2021). Tetracyclines in the Environment: An Overview on the Occurrence, Fate, Toxicity, Detection, Removal Methods, and Sludge Management. Sci. Total Environ..

[26] Lundström S.V., Östman M., Bengtsson-Palme J., Rutgersson C., Thoudal M., Sircar T., Blanck H., Eriksson K.M., Tysklind M., Flach C.-F. (2016). Minimal Selective Concentrations of Tetracycline in Complex Aquatic Bacterial Biofilms. Sci. Total Environ..

[27] Huang A., Yan M., Lin J., Xu L., Gong H., Gong H. (2021). A Review of Processes for Removing Antibiotics from Breeding Wastewater. Int. J. Environ. Res. Public Health.

[28] Menz J., Olsson O., Kümmerer K. (2019). Antibiotic Residues in Livestock Manure: Does the EU Risk Assessment Sufficiently Protect against Microbial Toxicity and Selection of Resistant Bacteria in the Environment?. J. Hazard. Mater..

[29] Yin G., Sun Z., Gao Y., Xu S. (2021). Preparation of Expanded Graphite for Malachite Green Dye Removal from Aqueous Solution. Microchem. J..

[30] Chen Q., Lua A.C. (2020). Kinetic Reaction and Deactivation Studies on Thermocatalytic Decomposition of Methane by Electroless Nickel Plating Catalyst. Chem. Eng. J..

[31] Gao Y., Zhu Y., Lyu L., Zeng Q., Xing X., Hu C. (2018). Electronic Structure Modulation of Graphitic Carbon Nitride by Oxygen Doping for Enhanced Catalytic Degradation of Organic Pollutants through Peroxymonosulfate Activation. Environ. Sci. Technol..

[32] Krawczyk P. (2011). Effect of Ozone Treatment on Properties of Expanded Graphite. Chem. Eng. J..

[33] Cychosz K.A., Thommes M. (2018). Progress in the Physisorption Characterization of Nanoporous Gas Storage Materials. Engineering.

[34] Nguyen T.T., Kim D.G., Ko S.O. (2022). Changes in the Catalytic Activity of Oxygen-Doped Graphitic Carbon Nitride for the Repeated Degradation of Oxytetracycline. Chemosphere.

[35] Li B., Zhang Y., Xu J., Mei Y., Fan S., Xu H. (2021). Effect of Carbonization Methods on the Properties of Tea Waste Biochars and Their Application in Tetracycline Removal from Aqueous Solutions. Chemosphere.

[36] Shafeeyan M.S., Daud W.M.A.W., Houshmand A., Shamiri A. (2010). A Review on Surface Modification of Activated Carbon for Carbon Dioxide Adsorption. J. Anal. Appl. Pyrolysis.

[37] Wang Z., Cao J., Wang J. (2009). Pyrolytic Characteristics of Pine Wood in a Slowly Heating and Gas Sweeping Fixed-Bed Reactor. J. Anal. Appl. Pyrolysis.

[38] Lee A.Y., Yang K., Anh N.D., Park C., Lee S.M., Lee T.G., Jeong M.S. (2021). Raman Study of D* Band in Graphene Oxide and Its Correlation with Reduction. Appl. Surf. Sci..

[39] Jaworski S., Wierzbicki M., Sawosz E., Jung A., Gielerak G., Biernat J., Jaremek H., Łojkowski W., Woźniak B., Wojnarowicz J. (2018). Graphene Oxide-Based Nanocomposites Decorated with Silver Nanoparticles as an Antibacterial Agent. Nanoscale Res. Lett..

[40] Yan H., Zhang M., Wang S., Li H., Kunsági-Máté S., Yin S. (2023). Raman Spectroscopy of Strained Monolayer Graphene Modulated by Monodispersed Au Nanoparticles. Appl. Surf. Sci..

[41] Zhang S., Liu Y., Gu P., Ma R., Wen T., Zhao G., Li L., Ai Y., Hu C., Wang X. (2019). Enhanced Photodegradation of Toxic Organic Pollutants Using Dual-Oxygen-Doped Porous g-C3N4: Mechanism Exploration from Both Experimental and DFT Studies. Appl. Catal. B Environ..

[42] Ghosh S., Ganesan K., Polaki S.R., Ravindran T.R., Krishna N.G., Kamruddin M., Tyagi A.K. (2014). Evolution and Defect Analysis of Vertical Graphene Nanosheets. J. Raman Spectrosc..

[43] Saha A., Basiruddin S.K., Ray S.C., Roy S.S., Jana N.R. (2010). Functionalized Graphene and Graphene Oxide Solution via Polyacrylate Coating. Nanoscale.

[44] Stevanović G., Mojović Z., Bogdanović D.B., Barudžija T., Jović-Jovičić N., Banković P., Ajduković M. (2025). The Influence of Preparation Method on the Efficiency of Clay-Carbon Composites for Detection of 4-Aminoantipyrine. Mater. Chem. Phys..

[45] Theodosiou A., Spencer B.F., Counsell J., Jones A.N. (2020). An XPS/UPS Study of the Surface/near-Surface Bonding in Nuclear Grade Graphites: A Comparison of Monatomic and Cluster Depth-Profiling Techniques. Appl. Surf. Sci..

[46] Pinder J.W., Major G.H., Baer D.R., Terry J., Whitten J.E., Čechal J., Crossman J.D., Lizarbe A.J., Jafari S., Easton C.D. (2024). Avoiding Common Errors in X-Ray Photoelectron Spectroscopy Data Collection and Analysis, and Properly Reporting Instrument Parameters. Appl. Surf. Sci. Adv..

[47] Liu Y., Jiang H., Liu C., Ge Y., Wang L., Zhang B., He H., Liu S. (2019). Influence of Functional Groups on Toxicity of Carbon Nanomaterials. Atmos. Chem. Phys..

[48] Fan C., Wei J., Huang H., Pan M., Fu Z. (2021). Chemical Feature of the Soot Emissions from a Diesel Engine Fueled with Methanol-Diesel Blends. Fuel.

[49] Abd A.A., Othman M.R., Kim J. (2021). A Review on Application of Activated Carbons for Carbon Dioxide Capture: Present Performance, Preparation, and Surface Modification for Further Improvement. Environ. Sci. Pollut. Res..

[50] Nwamba O.C., Echeverria E., McIlroy D.N., Austin A., Shreeve J.M., Aston D.E. (2019). Thermal Modification of Graphite for Fast Electron Transport and Increased Capacitance. ACS Appl. Nano Mater..

[51] Yang B., Kang H., Ko Y.-J., Woo H., Gim G., Choi J., Kim J., Cho K., Kim E.-J., Lee S.-G. (2021). Persulfate Activation by Nanodiamond-Derived Carbon Onions: Effect of Phase Transformation of the Inner Diamond Core on Reaction Kinetics and Mechanisms. Appl. Catal. B Environ..

[52] Komeily-Nia Z., Chen J.-Y., Nasri-Nasrabadi B., Lei W.-W., Yuan B., Zhang J., Qu L.-T., Gupta A., Li J.-L. (2020). The Key Structural Features Governing the Free Radicals and Catalytic Activity of Graphite/Graphene Oxide. Phys. Chem. Chem. Phys..

[53] Zou Q., Wang B., Gao B., Jiang T., Feng Q., Chen M., Zhang J., Zhang X. (2023). Roles and Mechanisms of Carbonaceous Materials in Advanced Oxidation Coupling Processes for Degradation Organic Pollutants in Wastewater: A Review. Biochar.

[54] Bellunato A., Arjmandi Tash H., Cesa Y., Schneider G.F. (2016). Chemistry at the Edge of Graphene. ChemPhysChem.

[55] Sharma R., Baik J.H., Perera C.J., Strano M.S. (2010). Anomalously Large Reactivity of Single Graphene Layers and Edges toward Electron Transfer Chemistries. Nano Lett..

[56] Zhu C., Zhang Y., Fan Z., Liu F., Li A. (2020). Carbonate-Enhanced Catalytic Activity and Stability of Co_3_O_4_ Nanowires for ^1^O_2_-Driven Bisphenol A Degradation via Peroxymonosulfate Activation: Critical Roles of Electron and Proton Acceptors. J. Hazard. Mater..

[57] Gao Y., Chen Z., Zhu Y., Li T., Hu C. (2020). New Insights into the Generation of Singlet Oxygen in the Metal-Free Peroxymonosulfate Activation Process: Important Role of Electron-Deficient Carbon Atoms. Environ. Sci. Technol..

[58] Liu J., Yao Z., Qiu G., Wan Y., Song W., Zeng H., Yang F., Zhao D., Yuan W., Ju P. (2023). Generation of Reactive Oxygen Species through Dissolved Oxygen Activation on Defected Porous Carbon for Efficient Degradation of Antibiotics. Chem. Eng. J..

[59] Yin R., Guo W., Wang H., Du J., Wu Q., Chang J.-S., Ren N. (2019). Singlet Oxygen-Dominated Peroxydisulfate Activation by Sludge-Derived Biochar for Sulfamethoxazole Degradation through a Nonradical Oxidation Pathway: Performance and Mechanism. Chem. Eng. J..

[60] Tian L., Chen P., Jiang X.-H., Chen L.-S., Tong L.-L., Yang H.-Y., Fan J.-P., Wu D.-S., Zou J.-P., Luo S.-L. (2022). Mineralization of Cyanides via a Novel Electro-Fenton System Generating ^−^OH and O_2_^•−^. Water Res..

[61] Huang B.-C., Jiang J., Huang G.-X., Yu H.-Q. (2018). Sludge Biochar-Based Catalysts for Improved Pollutant Degradation by Activating Peroxymonosulfate. J. Mater. Chem. A.

[62] Gao Y., Zhu Y., Chen Z., Zeng Q., Hu C. (2020). Insights into the Difference in Metal-Free Activation of Peroxymonosulfate and Peroxydisulfate. Chem. Eng. J..

[63] Murugan P., Nagarajan R.D., Shetty B.H., Govindasamy M., Sundramoorthy A.K. (2021). Recent Trends in the Applications of Thermally Expanded Graphite for Energy Storage and Sensors—A Review. Nanoscale Adv..

[64] Origins of Electron-Transfer Regime in Persulfate-Based Nonradical Oxidation Processes|Environmental Science & Technology. https://pubs.acs.org/doi/10.1021/acs.est.1c05374.

[65] Jiang Y., Gao K., Chen T., Xiong Y., Li Y., Addisu A., Pillai S.C., Dionysiou D.D., Wang D. (2024). Regulating the Generation of Singlet Oxygen (^1^O_2_) in Advanced Oxidation Processes by Catalyst Design for Water Treatment. Chem. Eng. J..

[66] Liang J., Fu L., Gao K., Duan X. (2022). Accelerating Radical Generation from Peroxymonosulfate by Confined Variable Co Species toward Ciprofloxacin Mineralization: ROS Quantification and Mechanisms Elucidation. Appl. Catal. B Environ..

[67] Yu Q., Ye J., Liu G., Liu M., Tang M., Li L. (2025). Exploring Electron-Transfer Pathways in Co-Pyrolyzed Waste-Derived Carbocatalyst for Enhanced Peroxydisulfate Activation. Sep. Purif. Technol..

[68] Li L., Chen N., An N., Feng C., Zheng Y., Zhao L., Li J., Zhang Z., Wang D., Cai Y. (2025). Persulfate Activation by Iron Complex: A Novel Non-Radical Strategy for Enhanced Tetracycline Degradation. Sep. Purif. Technol..

[69] Wu C., Zuo H., Du H., Zhang S., Wang L., Yan Q. (2022). Construction of Layered Embedding Dual Z-Scheme Bi_2_O_2_CO_3_/g-C_3_N_4_/Bi_2_O_3_: Tetracycline Degradation Pathway, Toxicity Analysis and Mechanism Insight. Sep. Purif. Technol..

[70] Ao X., Sun W., Li S., Yang C., Li C., Lu Z. (2019). Degradation of Tetracycline by Medium Pressure UV-Activated Peroxymonosulfate Process: Influencing Factors, Degradation Pathways, and Toxicity Evaluation. Chem. Eng. J..

[71] Xu A., Wei Y., Zou Q., Zhang W., Jin Y., Wang Z., Yang L., Li X. (2020). The Effects of Nonredox Metal Ions on the Activation of Peroxymonosulfate for Organic Pollutants Degradation in Aqueous Solution with Cobalt Based Catalysts: A New Mechanism Investigation. J. Hazard. Mater..

[72] Zhu J.-P., Lin Y.-L., Zhang T.-Y., Cao T.-C., Xu B., Pan Y., Zhang X.-T., Gao N.-Y. (2019). Modelling of Iohexol Degradation in a Fe(II)-Activated Persulfate System. Chem. Eng. J..

[73] Wang J., Wang S. (2021). Effect of Inorganic Anions on the Performance of Advanced Oxidation Processes for Degradation of Organic Contaminants. Chem. Eng. J..

[74] Huang J., Zhang H. (2019). Mn-Based Catalysts for Sulfate Radical-Based Advanced Oxidation Processes: A Review. Environ. Int..

[75] Jiang M., Lu J., Ji Y., Kong D. (2017). Bicarbonate-Activated Persulfate Oxidation of Acetaminophen. Water Res..

[76] Liang C., Wang Z.-S., Bruell C.J. (2007). Influence of pH on Persulfate Oxidation of TCE at Ambient Temperatures. Chemosphere.

[77] Zhu S., Wang Z., Ye C., Deng J., Ma X., Xu Y., Wang L., Tang Z., Luo H., Li X. (2022). Magnetic Co/Fe Nanocomposites Derived from Ferric Sludge as an Efficient Peroxymonosulfate Catalyst for Ciprofloxacin Degradation. Chem. Eng. J..

[78] Ma J., Li H., Chi L., Chen H., Chen C. (2017). Changes in Activation Energy and Kinetics of Heat-Activated Persulfate Oxidation of Phenol in Response to Changes in pH and Temperature. Chemosphere.

[79] Wang B., Wang Y. (2022). A Comprehensive Review on Persulfate Activation Treatment of Wastewater. Sci. Total Environ..

