# Supplementary material for: Dependency of Catalytic Reactivity on the Characteristics of Expanded Graphites as Representatives of Carbonaceous Materials
Source: Molecules. 2025 May 22;30(11):2275. doi: 10.3390/molecules30112275 (PMC12156407; doi:10.3390/molecules30112275)
Supplement: Supplementary file 1 [file molecules-30-02275-s001.zip › molecules-3624487-supplementary.pdf]

Supplementary information

Dependency of Catalytic Reactivity on the Characteristics of Expanded Graphites as Representatives of Carbonaceous Materials

Do Gun Kim[1,*], Seong Won Im[1,**], Kyung Hwan Ryu[2,***], Seoung Ho Jo[1], Min Gyeong Choe[1], and Seok Oh Ko[3,****]

[1] Department of Environmental Engineering, Sunchon National University, Suncheon, 57922, Republic of Korea
[2] Department of Chemical Engineering, Sunchon National University, Suncheon, 57922, Republic of Korea
[3] Department of Civil Engineering, Kyung Hee University, Yonggin, 17104, Republic of Korea

[*]Corresponding author: Do Gun Kim
Address: Department of Environmental Engineering, Sunchon National University, Suncheon, 57922, Republic of Korea
Phone: +82-61-750-3817
E-mail: dgkim@scnu.ac.kr
[**]Corresponding author: Seong Won Im
Address: Department of Environmental Engineering, Sunchon National University, Suncheon, 57922, Republic of Korea
Phone: +82-61-750-3818
E-mail: swim9828@scnu.ac.kr
[***]Corresponding author: Kyung Hwan Ryu
Address: Department of Chemical Engineering, Sunchon National University, Suncheon, 57922, Republic of Korea
E-mail: khryu@scnu.ac.kr
Phone: +82-61-750-3588
[****]Corresponding author: Seok Oh Ko
Address: Department of Civil Engineering, Kyung Hee University, 1732 Deokyoung-daero, Yongin-si, Gyeonggi-do, Republic of Korea
Phone: +82-31-201-2999
E-mail: soko@khu.ac.kr

This supplementary information contains 1 Text, 2 Tables, and 11 Figures.

Text S1. Quantitation of the contribution of reactive species by kinetic method.

The contribution of reactive species and electron transfer were calculated according to the following relationship (Eq. (S1)~(S8)).

$$kC_t = \left(k_{SO_4^{\bullet-}}\left[SO_4^{\bullet-}\right] + k_{^\bullet OH}\left[^\bullet OH\right] + k_{^1O_2}\left[^1O_2\right] + k_{e^-}\left[e^-\right]\right)C_t \quad \text{(S1)}$$

$$k_{SO_4^{\bullet-}}\left[SO_4^{\bullet-}\right] = k_{TBA} - k_{MeOH} \quad \text{(S2)}$$

$$k_{^\bullet OH}\left[^\bullet OH\right] = k - k_{TBA} \quad \text{(S1)}$$

$$k_{^1O_2}\left[^1O_2\right] = k - k_{L-his} \quad \text{(S3)}$$

$$k_{e^-}\left[e^-\right] = k - k_{KNO_3} \quad \text{(S4)}$$

$$f_{SO_4^{\bullet-}} = \frac{k_{SO_4^{\bullet-}}\left[SO_4^{\bullet-}\right]}{k_{SO_4^{\bullet-}}\left[SO_4^{\bullet-}\right] + k_{^\bullet OH}\left[^\bullet OH\right] + k_{^1O_2}\left[^1O_2\right] + k_{e^-}\left[e^-\right]} \quad \text{(S5)}$$

$$f_{^\bullet OH} = \frac{k_{^\bullet OH}\left[^\bullet OH\right]}{k_{SO_4^{\bullet-}}\left[SO_4^{\bullet-}\right] + k_{^\bullet OH}\left[^\bullet OH\right] + k_{^1O_2}\left[^1O_2\right] + k_{e^-}\left[e^-\right]} \quad \text{(S6)}$$

$$f_{^1O_2} = \frac{k_{^1O_2}\left[^1O_2\right]}{k_{SO_4^{\bullet-}}\left[SO_4^{\bullet-}\right] + k_{^\bullet OH}\left[^\bullet OH\right] + k_{^1O_2}\left[^1O_2\right] + k_{e^-}\left[e^-\right]} \quad \text{(S7)}$$

$$f_{e^-} = \frac{k_{e^-}\left[e^-\right]}{k_{SO_4^{\bullet-}}\left[SO_4^{\bullet-}\right] + k_{^\bullet OH}\left[^\bullet OH\right] + k_{^1O_2}\left[^1O_2\right] + k_{e^-}\left[e^-\right]} \quad \text{(S8)}$$

The k is the reaction rate constant in Eq. (3) and C_t is the TC concentration at time t. $\left[SO_4^{\bullet-}\right]$, $\left[^\bullet OH\right]$, $\left[^1O_2\right]$, and $\left[e^-\right]$ are the concentrations, $k_{SO_4^{\bullet-}}$, $k_{^\bullet OH}$, $k_{^1O_2}$, and k_{e^-} are the TC removal rate constant, $f_{SO_4^{\bullet-}}$, $f_{^\bullet OH}$, $f_{^1O_2}$, and f_{e^-} are the contributions, for $SO_4^{\bullet-}$, $^\bullet OH$, $^1O_2$, and electron transfer, respectively. The k_{MeOH}, k_{TBA}, k_{L-his}, and k_{KNO_3} are the k's in the presence of MeOH, TBA, L-his, and KNO_3, respectively.

Table S1. The components of XPS survey spectra.

		Peak BE (eV)	FWHM (eV)	Area (CPS*·eV)	Atomic %
EG	C1s	283.86	0.79	88405.82	88.8
	O1s	531.40	2.49	28117.36	11.2
EG350	C1s	283.95	0.81	96235.95	93.6
	O1s	532.59	2.84	16504.24	6.4
EG550	C1s	283.37	0.80	107508.90	96.0
	O1s	531.28	2.74	11245.43	4.0
EG550	C1s	284.8	0.81	834050.63	96.7
	O1s	532.93	2.97	70438.22	3.3

Note. *: Counts per second.

Table S2. Degradation products of tetracycline.

Products	m/z	Molecular formula	Chemical structure
Tetracycline	445	$C_{22}H_{24}N_2O_8$	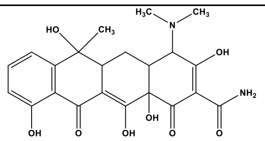
BP1	461	$C_{22}H_{24}N_2O_9$	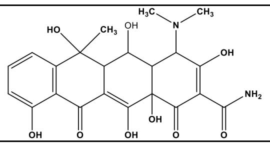
BP2	477	$C_{22}H_{24}N_2O_{10}$	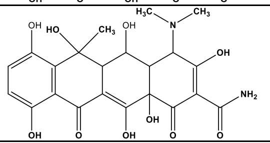
BP3	495	$C_{22}H_{26}N_2O_{11}$	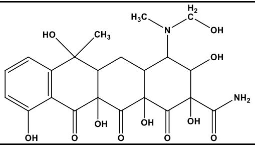
BP4	297	$C_{17}H_{12}O_5$	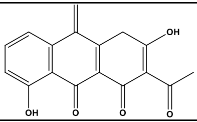
BP5	266	$C_{14}H_{18}O_5$	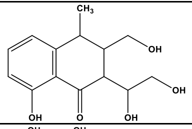
BP6	230	$C_{12}H_{22}O_4$	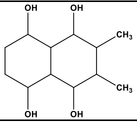
BP7	157	$C_8H_{12}O_3$	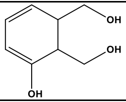
BP8	225	$C\text{-}H_{16}O_7$	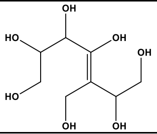
BP9	118	$C_4H_6O_4$	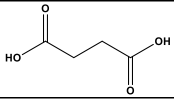

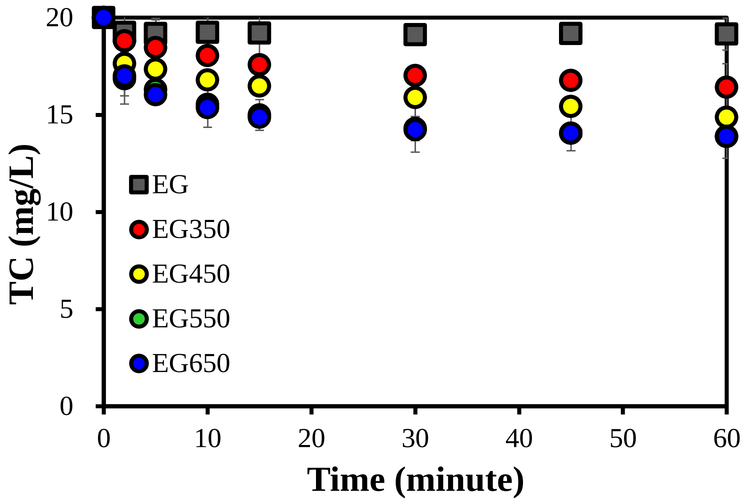

Figure S1. TC removal by adsorption onto EGs (EGs 0.1 g, TC 20 mg/L).

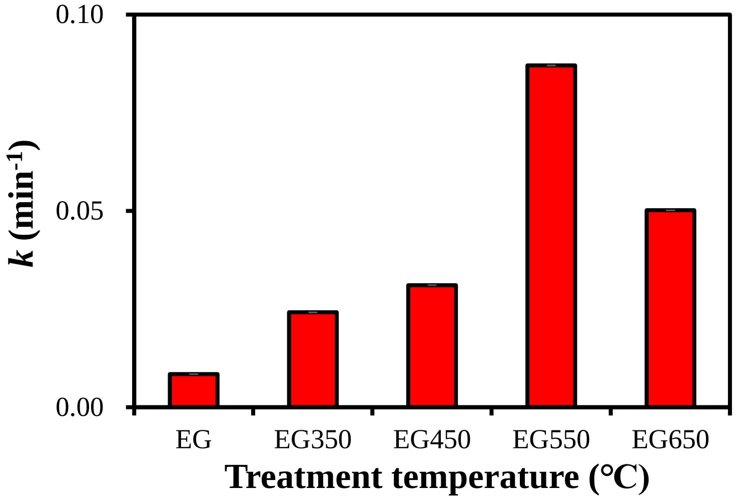

Figure S2. TC removal rate constants of EGs (EGs 0.1 g, PDS 0.1 mM, TC 20 mg/L).

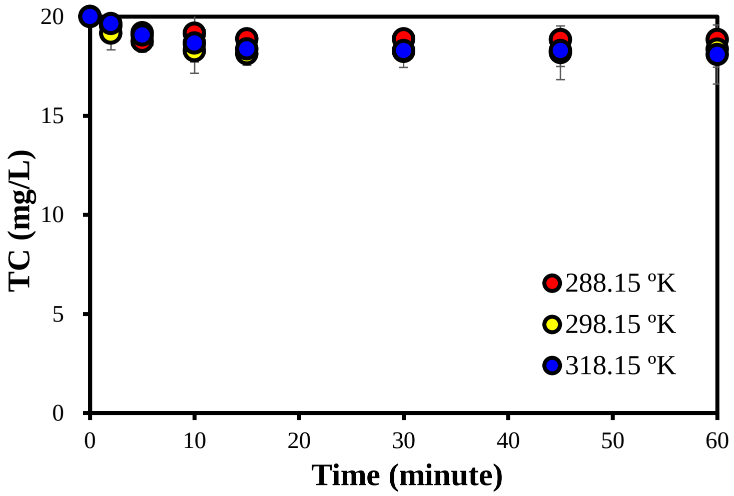

Figure S3. Effects of temperature on TC removal in EG+PDS (EG 0.1 g, PDS 0.1 mM, TC 20 mg/L).

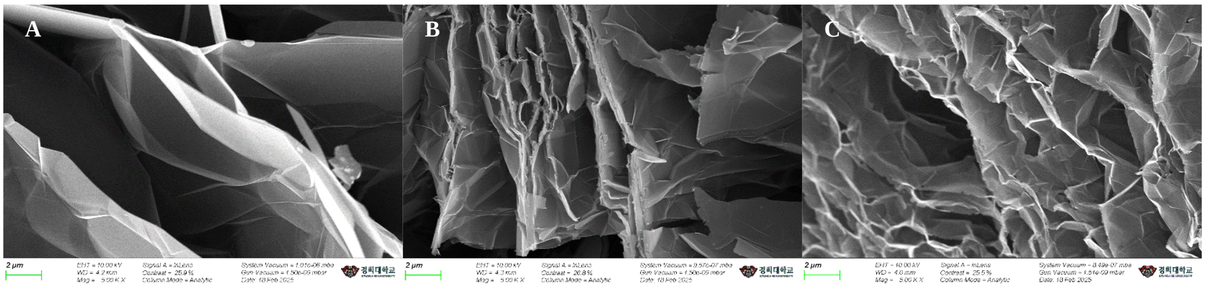

Figure S4. SEM images of (A) EG, (B) EG350, and (C) EG550.

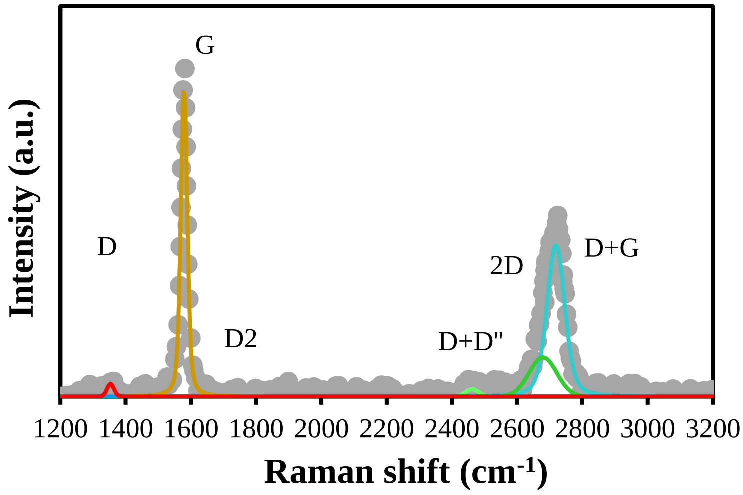

Figure S5. Raman spectrum of EG650.

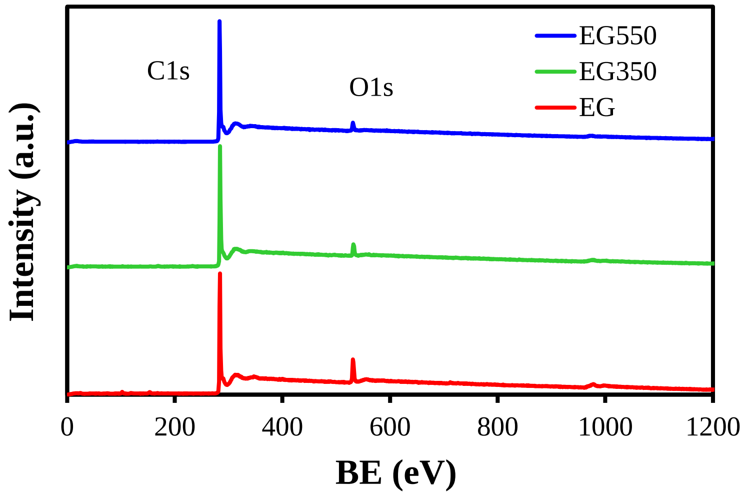

Figure S6. The XPS survey spectra of EG, EG350, and EG550.

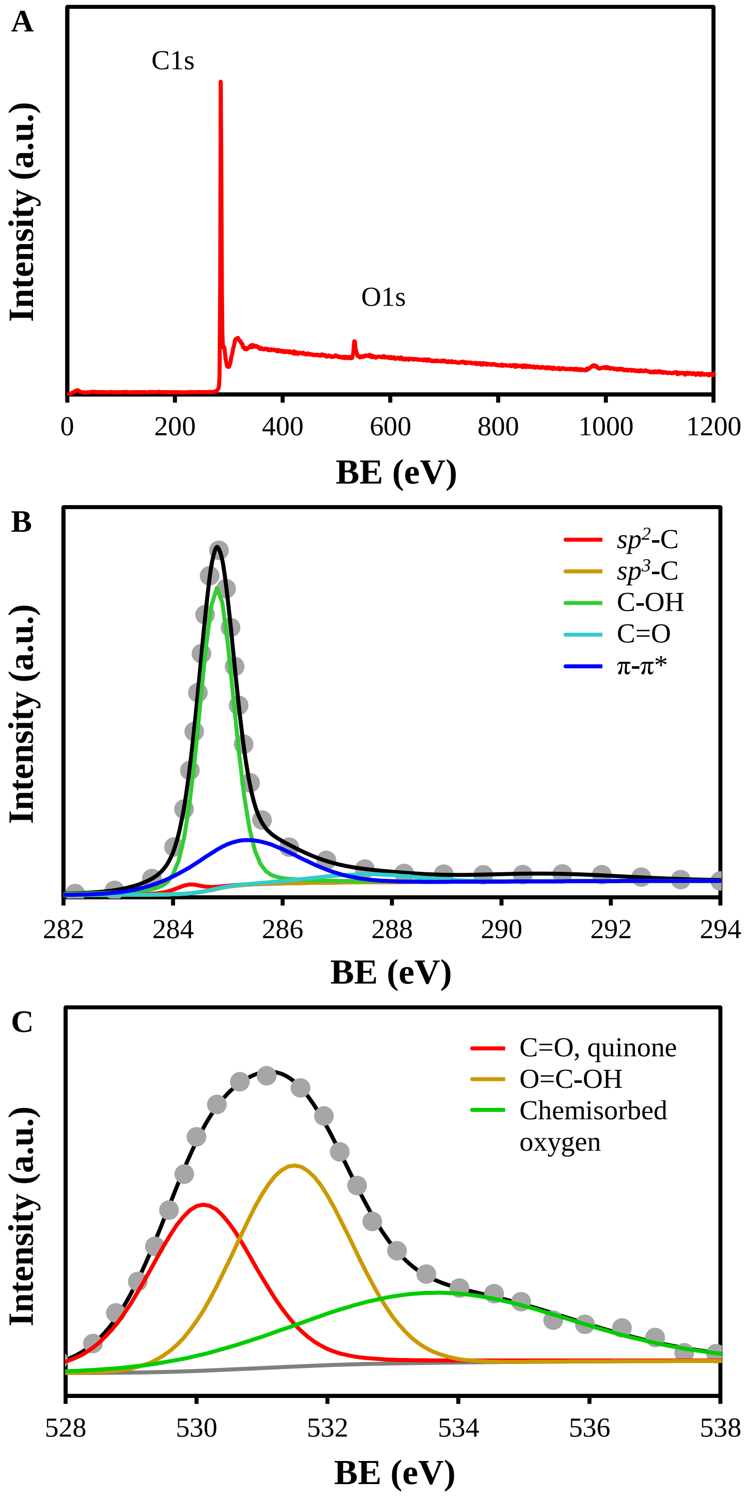

Figure S7. (A) Survey spectrum and high resolution spectra of (C) C1s and (C) O1s of EG650.

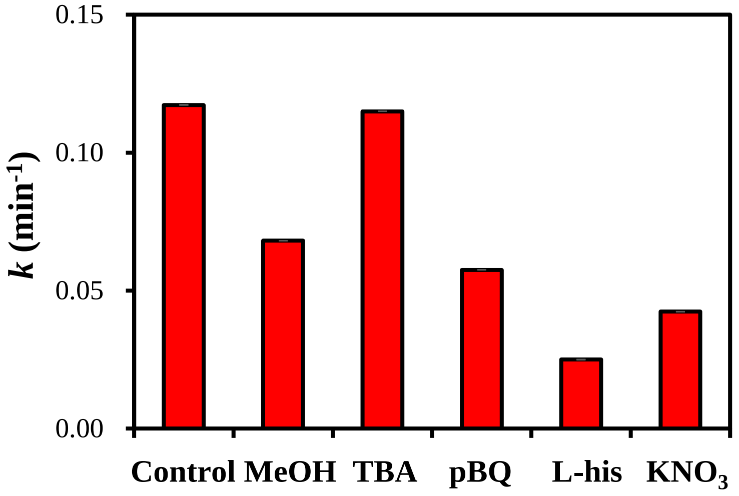

Figure S8. TC removal rate constant (k) in Control and in the presence of scavengers (EG550 0.1 g, TC 20 mg/L, PDS 0.1 mM).

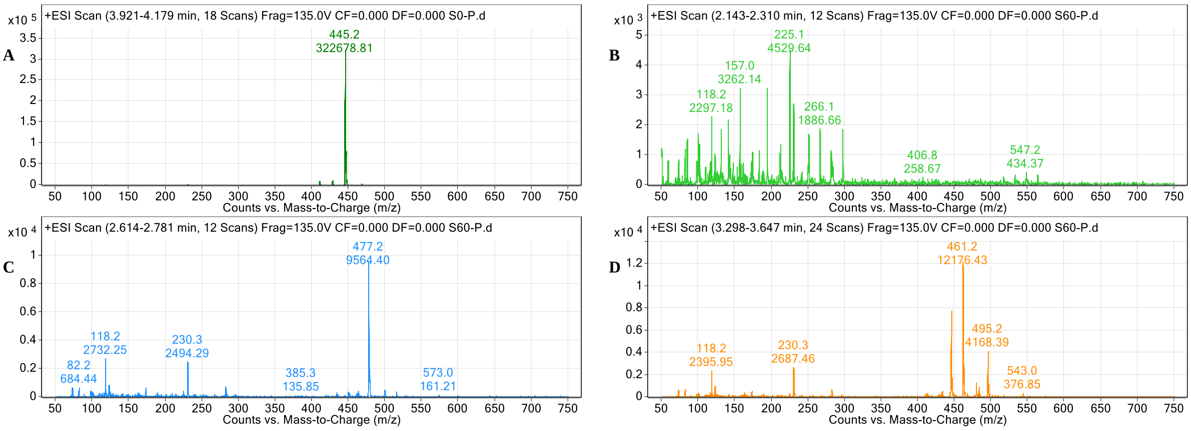

Figure S9. HPLC-MS/MS spectra of (A) TC and (B)~(D) the TC degradation intermediates in EG550+PDS at 60 minutes of reaction time.

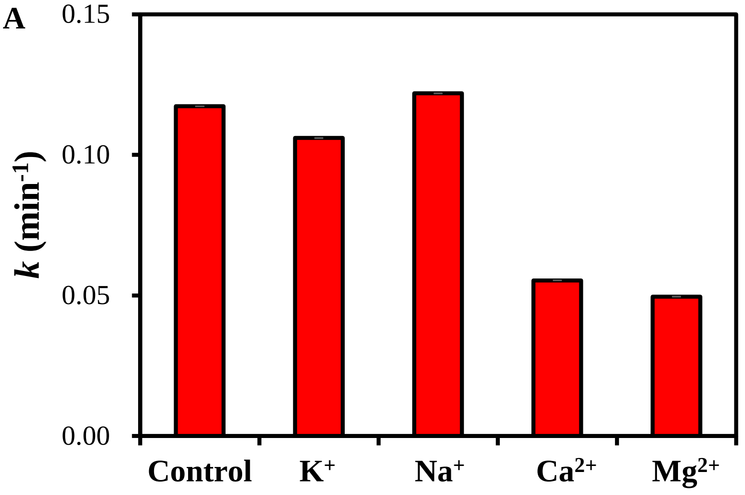

Figure S10. TC removal rate constant (k) in Control and in the presence of 10 mM cations (EG550 0.1 g, TC 20 mg/L, PDS 0.1 mM).

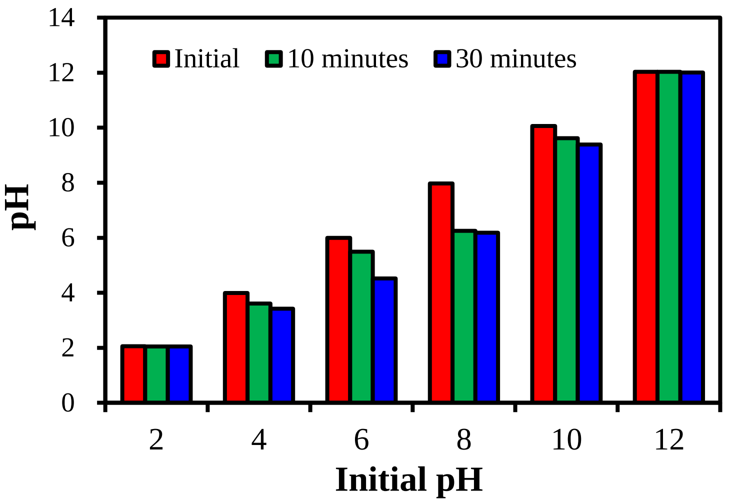

Figure S11. Changes in pH in EG550+PDS at different initial pH (EG550 0.1 g, TC 20 mg/L, PDS 0.1 mM).
